# GLP-1 receptor agonists in the context of cancer: the road ahead

**DOI:** 10.1152/ajpcell.00245.2025

**Published:** 2025-04-26

**Authors:** Isabelle R. Miousse

**Affiliations:** Department of Biochemistry and Molecular Biology, University of Arkansas for Medical Science, Little Rock, Arkansas, United States

**Keywords:** cachexia, cancer, GLP-1 receptor agonists, type 2 diabetes

## Abstract

A rapidly increasing proportion of the population in the United States is taking glucagon-like peptide-1 receptor agonists (GLP-1RAs) for type 2 diabetes or weight loss. Consequently, an increasing number of patients presenting with new cases of cancer also have a current prescription for GLP-1RAs. The impact of GLP-1RAs on metabolism is quite profound, and it is entirely reasonable to assume these agents are also very impactful on the metabolism of cancer cells, in addition to the general metabolism of the patient. Although these drugs are relatively recent on the market, the study of metabolism in cancer is a well-established field and we can make predictions about how GLP-1RAs will interface with cancer treatments. In fact, some evidence points to a possible neoadjuvant effect of these drugs for patients with cancer that would justify the initiation of GLP-1RAs to support therapy in a subset of patients. At the same time, there is a very present concern that drugs that induce weight loss may also precipitate the loss of muscle mass, cachexia, in patients. Here, we will provide an overview of the existing literature around diabetes and metabolism in the context of cancer and cachexia.

## WHAT ARE GLP-1RAs?

Glucagon-like peptide-1 receptor agonists (GLP-1RAs) are synthetic analogs of the glucagon-like peptide-1 (GLP-1) hormone. The endogenous peptide hormone GLP-1 binds to the GLP-1 receptor and regulates glucose levels and satiety signals. This function makes it an excellent target for the treatment of type 2 diabetes (T2D) and obesity. Efforts to synthesize GLP-1RAs for clinical applications date back to the 1990s (1). The endogenous GLP-1 hormone is rapidly degraded by the enzyme dipeptidyl peptidase-4 (DPP-4), resulting in a half-life of less than 5 min in the circulation (2, 3). Work to increase the lifespan of GLP-1RAs led to the first compound approved by the Food and Drug Administration (FDA), exenatide (Byetta), in 2005 (4–6). Exenatide was derived from the exendin-4 protein, a functional analog of GLP-1, isolated from Gila monster venom (7). The variations found in the Gila monster peptide sequence led to an improved half-life of 2 to 3 h in circulation (8). The addition of a fatty acid side chain led to the approval of liraglutide, with a half-life of ~13 h, in 2010 (9). Further increasing albumin binding properties then led to a half-life of 1 wk after injection for semaglutide (10). Fusion of GLP-1 to a modified Fc fragment of human immunoglobulin G4 also led to a variant GLP-1RA with a similar half-life of ~1 wk, dulaglutide (11). Finally, semaglutide is now available as a once daily oral medication thanks to the addition of an absorption enhancer to the formulation (12). The GLP-1RA doses used by patients with T2D are lower than for weight loss, but they will still typically experience an ~2.5% reduction of their body weight (13).

The main GLP-1RAs currently on the market are semaglutide (Ozempic, Wegovy, and Rybelsus), dulaglutide (Trulicity), as well as tirzepatide (Mounjaro and Zepbound). Tirzepatide is a dual agonist for the glucose-dependent insulinotropic polypeptide receptor (GIPR) and the GLP-1 receptor (14, 15). Semaglutide was approved by the FDA on December 5, 2017. By 2023, the combined annual sales for semaglutide alone were estimated at $US 21.1 billion worldwide, and $US 15.0 billion in the United States (16). Tirzepatide was approved in mid-2022 and is following a similar upward trend. Finally, the triple agonist retatrutide (GIPR, GLP-1 receptor, and glucagon receptor) is currently under clinical investigation with promising early results (17). Recent polls suggest that 5%–8% of adults in the United States use GLP-1RAs (18–20), despite high costs and drug shortages. Given that both T2D and cancer incidence increase with age, the proportion of patients with cancer using GLP-1RAs is almost certainly higher.

## MODE OF ACTION

GLP-1RAs have had a transformational impact on the management of T2D since the approval of semaglutide in late 2017. The GLP-1 receptor is expressed mainly on pancreatic β cells. In the presence of glucose, this binding stimulates the release of insulin and suppresses glucagon secretion, improving glucose uptake in the periphery (reviewed in Ref. 21). GLP-1 may also directly stimulate glucose uptake in muscle cells (22). In nonhuman primates, expression of the GLP-1 receptor was detected in regions of the brain involved in the regulating food intake (23). The presence of the receptor in the brain would also explain the apparent effects of GLP-1RAs in curbing addictive behaviors (24–26). GLP-1RAs were shown to slow gastric emptying (27–29), an effect that is thought to be due to a direct action on the smooth muscle cells of the gastrointestinal tract (30). The delay in gastric emptying contributes both to a desirable reduction in food intake and to side effects like nausea, which occurs in ~20% of patients and leads to discontinuation in just over 1% of patients (15). As a result of these combined effects, most patients with T2D using GLP-1RAs experience an improvement in fasting serum insulin and insulin sensitivity (homeostasis model assessment for insulin resistance), a lowering of hemoglobin A1c levels, and a decreased reliance on supplemental insulin (12, 31).

## DIABETES AND CANCER

Overall cancer incidence is markedly higher in patients with T2D, particularly for cancers of the gastrointestinal tract (32). Based on epidemiological data, chronic hyperglycemia in poorly controlled patients with T2D is thought to have a cancer-promoting proliferative effect (reviewed in Ref. 33). Chronically elevated inflammation and circulating insulin are two additional characteristics of T2D that have been proposed to promote tumor growth (33). It therefore makes sense that risk would be lowered by promoting better control of blood glucose levels in these individuals. The data in fact indicate that patients with T2D who receive GLP-1RAs and no longer require insulin had a lower incidence of cancers of the gastrointestinal tract compared with patients with T2D treated with supplemental insulin and no GLP-1 agonists (34). The data suggest that GLP-1RAs may neutralize the T2D-induced increase in cancer incidence.

Of note, an increase in thyroid cancer incidence has been reported with the use of GLP-1RAs in patients with T2D (35). However, this result is debated (36, 37). Similarly, GLP-1RAs are associated with an increase in the incidence of pancreatitis (38, 39). However, it is still unclear whether this affects the incidence of pancreatic cancer (40, 41).

In addition to having a higher cancer incidence, patients with T2D have been shown to have poorer cancer prognosis than patients without T2D (42–44). Patients with cancer who present with preexisting T2D have higher odds of negative outcomes such as cachexia, recurrence, and all-cause mortality than patients without diabetes. The data also suggest that patients with T2D are treated less aggressively for their cancer, possibly linked to more frequent treatment interruptions (45). Consequently, removing the risk factors associated with T2D in patients with cancer with GLP-1RAs may not only cancel the elevated cancer incidence but also improve survival. By presenting a better metabolic profile, patients using GLP-1RAs may be in a better position to respond to therapy and manage side effects. In fact, the summation of the data presented here suggests that this would be the case in patients with T2D, but it also points to a potential benefit for patients with cancer who may not have a current diagnosis of T2D. Consequently, there may be a benefit to using GLP-1RA therapy as a neoadjuvant to promote an improved response in at least a subset of patients with cancer.

## THE METABOLIC RAMIFICATIONS OF CALORIC RESTRICTION IN CANCER

To understand the activity of GLP-1RAs in healthy individuals and in patients with T2D, it is helpful to use caloric restriction (CR) as a reference. CR, or the reduction of daily caloric intake without malnutrition (~20%), has been repeatedly associated with a decrease in cancer incidence in animal models since the very early 20th century (46), including in primates (47, 48). As we have presented earlier, GLP-1RAs reduce food intake (i.e., a form of spontaneous CR) and are associated with lower cancer incidence. This provides at least a superficial similarity between the two approaches. The decades of research on CR suggest that the reduction in cancer incidence in CR is facilitated by the reduction in glucose, insulin, and IGF-1 levels, which would otherwise stimulate cell growth and proliferation, and the reduction in oxidative stress and inflammation (49, 50). Once again, we see an overlap between CR and the metabolic effects of GLP-1RAs. Notably, CR reduces not only cancer incidence but also tumor growth and metastasis in animals (49, 51). Similarly, reducing dietary intake specifically of the amino acid methionine reduces insulin, IGF-1, oxidative stress, and inflammation, and reduces tumor growth in animal models (52–54). Exercise has overlapping effects with CR in enhancing insulin sensitivity and decreasing inflammation (55), and has been associated with lower cancer incidence (56) and reduced tumor growth (57). The data available indicate that CR and related interventions create a metabolic environment less favorable to the initiation and progression of cancer. This suggests that similar effects may be found associated with the use of GLP-1RAs.

## FROM DIABETES TO CANCER CARE

In further support of this hypothesis, animal studies now suggest that GLP-1RAs reduce tumor growth. Interestingly, the data supporting the use of GLP-1RAs come from normoglycemic animals. At the time of writing, we have identified six peer-reviewed studies where GLP-1RAs were used as cancer therapy in an animal tumor model. Tirzepatide (dual GIP/GLP-1RA) was administered to mice in which endometrial tumors had been induced with a cytomegalovirus promoter (58). Whether the animals consumed a high-fat diet or not, tirzepatide administration significantly reduced tumor weight. Another group found a decrease in tumor growth in a xenograft transplantation model of oral squamous cell carcinoma (OSCC) with semaglutide administration (59). A similar decrease in tumor volumes with semaglutide was found in a syngeneic orthotopic model of breast cancer, along with a decrease in metastases to the liver (60). The triple agonist retatrutide was also found to reduce the growth of tumors in a normoglycemic obese model of triple negative breast cancer (61). In that study, there was a significant benefit to combining retatrutide with the chemotherapeutic compound gemcitabine in female mice. Retatrutide was found to reduce tumor volume in a pancreatic cancer model (62). In that study, semaglutide and CR significantly reduced tumor volumes, but the triple agonist showed greater differences from control volumes. The authors showed an improvement in markers of antigen presentation with retatrutide. Semaglutide failed to provide benefit for either tumor growth or antigen presentation when it was discontinued prior to tumor cell injection in a metastatic melanoma model (63). This model suggests that antitumor effects may be dependent on concurrent administration and not mediated solely by weight loss. We identified two additional studies that were not yet peer-reviewed at the moment of writing. These studies suggest that tirzepatide reduce the growth of breast (64) and colorectal (65) tumors in a diet-induced obese model. One of these teams measured lean mass in their model and reported that it was unchanged by tirzepatide (65).

Although two of the studies are not yet peer-reviewed, nascent preclinical research is providing preliminary indication that GLP-1RAs may have a positive impact in reducing cancer growth. It is not entirely clear whether GLP-1RAs have direct effects on tumor cells, or if the benefits seen in animal models are entirely due to the indirect effect of decreased food intake. Expression of the GLP-1 receptor is found at a high level in most benign insulinomas (66). Expression in other tumor types, including malignant insulinomas, appears to be variable (reviewed in Ref. 67). Among the studies listed in the previous paragraph, Kong et al. (58) reported that GLP-1 receptor gene expression was detected in endometrial tumors and that expression was higher in lean mice and reduced by tirzepatide. Protein expression of the GLP-1 receptor was also found to be present at higher levels in OSCC tissue than in normal tissues (59). Semaglutide decreased migration, proliferation, and viability in OSCC cells in a dose-dependent manner (59), suggesting a direct-action mechanism. However, it remains uncertain if expression of the GLP-1 receptor is associated with the tumor response in vivo. It appears that the question of direct versus indirect effects cannot be answered with the existing evidence. Finally, at the moment of writing, one human clinical study reported that patients with lung cancer and T2D showed a significantly improved prognosis when cotreated with a combination of sodium-glucose cotransporter 2 inhibitors and GLP-1RAs (68).

## CACHEXIA: THE OVERLOOKED COMPLICATION IN CANCER

Although the rationale supporting the use of GLP-1RAs in cancer is compelling, this discussion would be incomplete without addressing the problem of cachexia. Cachexia is a significant loss of body weight, mainly from muscle mass, that induces a loss of physical function and cannot be reversed by conventional nutritional means (69). Cachexia is directly responsible for up to a third of cancer-related deaths and is a contributing factor in a much larger proportion (70).

The underlying causes of cachexia remain poorly understood. Some tumors trigger a hypermetabolic state where muscle is catabolized in an uncontrolled manner. Inflammation is known to be tightly associated with the development of cachexia (69). Cachexia significantly weakens patients who become less able to tolerate therapies, leading to discontinuation of life-saving treatments. Although some management options do exist, there is a critical need for new treatments to manage and reverse cachexia in patients with cancer.

## GLP-1 AGONISTS IN THE CONTEXT OF CACHEXIA

**Figure 1.**
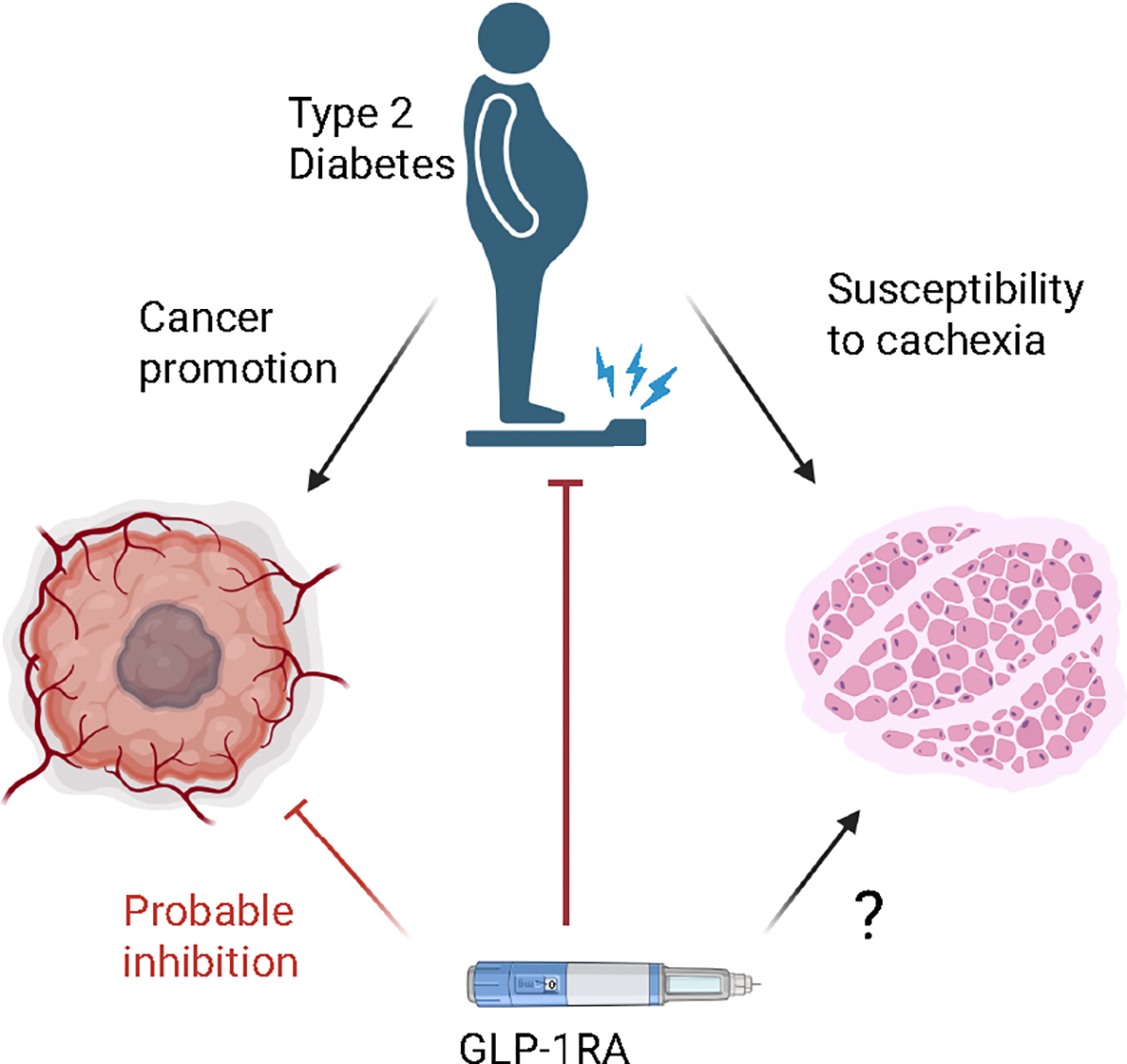
In patients with cancer, T2D promotes cancer progression (*left*) and increases susceptibility to muscle loss (*right*). GLP-1RAs (*bottom*) probably have an inhibitory effect on cancer progression, but their effect on muscle is unknown. Figure created with a licensed version of Biorender. com. GLP-1RAs, glucagon-like peptide-1 receptor agonists; T2D, type 2 diabetes.

There is an entirely justified concern that GLP-1RAs, by suppressing food intake, may worsen or even trigger cachexia in patients with cancer (Fig. 1). The appetite-suppressing and weight-loss properties of GLP-1RAs appear in direct contradiction with the need to maintain muscle mass throughout cancer treatments. It is worthwhile to review the effects of GLP-1RAs to evaluate the potential advantages and disadvantages, identify our gaps in knowledge, and recognize where the information to fill these gaps may come from.

GLP-1RAs induce weight loss by reducing food intake (71–74). Some of that loss is in the form of muscle mass. A closer look at the large amount of data available on the semaglutide-associated weight loss points to several relevant facts. First, fat mass loss far outweighs lean mass loss in individuals using GLP-1RAs. In fact, lean mass relative to total body mass increases on average in these individuals (75). Some patients do not experience any loss in muscle mass, whereas others do show more loss in muscle. The data suggest that the decrease in lean mass is comparable to that seen with bariatric surgery, and exercise capacity generally improves following bariatric surgery (76). We should also note that both for GLP-1RAs and bariatric surgery, the loss of fat mass is much improved when combining treatment with exercise. As exercise itself is suggested to have positive effects on clinical outcomes including survival (77), the combination of GLP-1RAs and exercise in patients with cancer appears to be a viable option to both promote response to cancer treatments and protect muscle mass.

As previously mentioned, an important common aspect of cancer cachexia is the presence of inflammation (69). GLP-1RAs have been shown to decrease systemic inflammation (78). The implication is that GLP-1 agonists may actively decrease the risk of cachexia in cancer by mitigating the associated inflammatory environment. Animal studies support this hypothesis, showing lower levels of inflammation and improved muscle fiber ratio, area, and density in obese mice administered semaglutide (79). In addition, some evidence points to the expression of the GLP-1 receptor in skeletal myocytes, with possible direct effects of GLP-1RAs on skeletal muscles (22). There is currently no evidence pointing definitively one way or another when it comes to the effect of GLP-1RAs on muscle mass in patients with cancer, and both alternatives are reasonable viewpoints until studies provide clearer evidence. Such studies are continuing for patients without cancer (NCT05786521, NCT06811324, and NCT06497595) (80) and are being initiated in the context of cancer (NCT06751589). Additional ongoing clinical trials of GLP-1RA in patients with cancer do not specifically mention muscle mass as an end point (NCT06518837 and NCT06073184).

## AN ONGOING EXPERIMENT

The number of patients with cancer presenting at diagnosis with a preexisting GLP-1RAs prescription is rapidly increasing, along with the proportion of the total adult population using these agents. The principle of precaution may suggest a discontinuation of the medication when taken for weight loss. However, when prescribed for T2D, the same principle of precaution may warrant the maintenance of an effective strategy to control an active disease while undergoing cancer treatments. This represents a vast and growing population of patients for which there is a solid incentive to combine GLP-1RAs with cancer treatments, not by choice but out of necessity.

How will these GLP-1RAs patients with cancer compare with others with T2D? Many confounding elements will factor in this natural experiment, such as residual insulin use, exercise, frailty score, nutritional status, and socioeconomic status. It is our responsibility to realize the magnitude of this sudden change in clinical management of T2D. There is a critical need to gather the data necessary for future cohorts of patients and physicians to make informed choices when it comes to continuing or discontinuing these medications during treatment.

Will GLP-1RAs precipitate cancer cachexia? Or will they improve response to therapy? Does the answer depend on preexisting patient-related factors that we are unaware of as of now? Ultimately, the integration of GLP-1RAs into cancer care is already happening. We need the data to understand how to reap the largest benefits out of these powerful metabolic modulators to promote both survival and quality of life in patients with cancer.
